# Supporting community health workers in South Africa for context-specific food and nutrition literacy: implementation of a multi-media education-entertainment intervention

**DOI:** 10.1186/s40795-025-01124-z

**Published:** 2025-07-25

**Authors:** Elochukwu C. Okanmelu, Machoene D. Sekgala, Peter Delobelle, Olufunke Alaba, Nicole Holliday, Jillian Hill, Martina Lembani, Zandile J. Mchiza

**Affiliations:** 1 Institute for Medical Information Processing, Biometry and Epidemiology (IBE), Chair of Public Health and Health Services Research, Faculty of Medicine, LMU Munich, Elisabeth-Winterhalter-Weg 6, D-81677 Munich, Germany; 2 Pettenkofer School of Public Health, Munich, Germany; 3 Non-Communicable Disease Research Unit, South African Medical Research Council, Francie van Zijl Drive, Parowvallei, Tygerberg, 7505 Cape Town, Western Cape South Africa; 4 Department of Social Sciences, Center for Social Sciences Research (CSSR), University of Cape Town, Robert Leslie Social Science Building 12 University Avenue, 7701 Cape Town, Western Cape South Africa; 5 Chronic Diseases Initiative for Africa, University of Cape Town, 7925 Cape Town, Western Cape South Africa; 6 Department of Public Health, Vrije Universiteit Brussel, Laarbeeklaan 101, 1090 Brussels, Belgium; 7 School of Public Health, University of the Western Cape, Robert Sobukwe Road, 7535 Cape Town, Western Cape South Africa

**Keywords:** Multimedia education-entertainment, Food and nutrition literacy, Community health workers, Malnutrition, South Africa

## Abstract

**Background:**

Multimedia technology, recognized for its efficacy in education, offers a complementary approach to traditional health education. In South Africa, community health workers (CHWs) play a pivotal role in improving population health, but often lack comprehensive health knowledge. This study explores the effectiveness of using a multimedia education-entertainment (MM-EE) intervention to enhance food and nutrition literacy among CHWs in resource-challenged townships.

**Methods:**

Seventy-seven participants completed a baseline and 6-month post-intervention follow-up survey. The MM-EE intervention comprised short stories conveyed via comic booklets and 30-second video clips, covering food knowledge, use of food labels, meal preparation, planning, and eating behaviour. Materials were distributed via mobile messaging services, including WhatsApp (as the primary social media platform), MMS, and SMS. Descriptive statistics, chi-square tests, Wilcoxon signed-rank tests, and multivariate linear regression were conducted using R studio version 4.3.3.

**Results:**

Over 70% of participants were classified as having obesity, and 18.2% as overweight, leading to 88.3% of participants being classified as overweight or having obesity. A significant proportion of participants (68.8%) reported having an existing non-communicable disease (NCD). The MM-EE intervention significantly improved CHWs’ food and nutrition literacy, reflected in improved median scores for meal preparation (43.1% [IQR: 19.6] pre- vs. 62.7% [IQR: 11.7] post-intervention), planning (58.6% [IQR: 12.1] pre- vs. 67.2% [IQR: 13.4] post-intervention), and overall food and nutrition literacy (63.0% [IQR: 8.1] pre- vs. 69.4% [IQR: 10.9] post-intervention; *p* <.001).

**Conclusion:**

The study showed effective MM-EE intervention outcomes, positioning this method of health dissemination as suitable for improving the food and nutrition literacy of CHWs in South Africa. The study suggests the potential effectiveness of MM-EE approaches to enhance the health knowledge of the South African population. However, adaptation for the wider population will require further research on scalability, sustainability and an improved intervention design to address all dimensions of literacy.

**Supplementary Information:**

The online version contains supplementary material available at 10.1186/s40795-025-01124-z.

## Key message

High levels of food and nutrition literacy (FNL) are key to preventing diet-related health issues and improving health outcomes and overall well-being globally. CHWs play a pivotal role in bridging the gap between healthcare services and communities, making them ideal agents for disseminating nutrition information and fostering behaviour change. This intervention study aimed to increase food and nutrition literacy among community health workers in resource limited townships in South Africa, with the potential to enable food and nutrition-related health education of the communities they serve. The effectiveness of this study further signals the potential of MM-EE in improving population literacy.

**Supplementary Information:**

The online version contains supplementary material available at 10.1186/s40795-025-01124-z.

## Introduction

In a shifting landscape characterized by intricate health challenges and dynamic dietary trends, the enhancement of food and nutrition literacy (FNL) is important to ensuring improved health outcomes and overall well-being [1, 2]. This is particularly the case in South Africa, a country that faces a double burden of malnutrition (DBM), the syndemic coexistence of overnutrition, undernutrition, and micronutrient deficiencies [3].

While the definitions of food literacy and nutrition literacy intersect on a theoretical level, standard universal definitions are still absent [4–6]. Nutrition literacy is often defined as “the extent to which individuals have the capacity to access, process, understand, and implement nutritional information and skills necessary to make appropriate dietary decisions” on functional, interactive, and critical levels [1, 5, 7, 8]. While functional nutrition literacy involves acquiring, comprehending, and utilizing nutrition information, interactive nutrition literacy pertains to engaging in dietary communication and discussion, and critical nutrition literacy encompasses evaluating nutritional data and comprehending food-environment connections [4]. Food literacy is defined as the comprehensive set of skills and abilities essential for effectively planning, managing, selecting, preparing, and consuming food, comprising 11 components categorized into four domains: planning and managing, selecting, preparing, and eating [9, 10]. These skills not only aid individuals’ autonomy in food choices but also foster critical capacities in food selection and consumption, while facilitating an understanding of the broader implications of individual and communal food-related decisions on society, health, and the environment [8, 9]. Using the above definitions, food and nutrition literacy (FNL) not only entails the knowledge of food and nutrition but also the perception of, and practical application of these knowledge and skills [4, 5, 7, 9]. FNL, therefore, is understood as an integrated construct that encompasses both nutrition literacy and food literacy. FNL refers to an individual’s combined knowledge, skills, and capacities to access, understand, evaluate, and apply information related to food and nutrition. It includes not only the ability to make informed dietary choices based on nutritional knowledge but also the practical skills involved in planning, selecting, preparing, and consuming food. Importantly, FNL involves critical awareness of the broader social, health, and environmental contexts of food-related decisions, supporting autonomy and empowerment in fostering healthier and more sustainable food practices [4–9]. Addressing the multifaceted challenges of the malnutrition syndemic necessitates equipping individuals with skills to choose, prepare and consume, and preserve food wisely. Hence, the need for innovative and scalable strategies that can effectively reach diverse populations, particularly those in resource-constrained settings.

Multi-media education-entertainment (MM-EE) materials have emerged as promising tools for enhancing knowledge and facilitating behavioural change in health-related interventions [11–13]. MM-EE approaches, utilizing a spectrum of media channels such as radio, television, social media, games and mobile applications, have demonstrated the capacity to overcome literacy barriers, reaching individuals across different age groups and educational backgrounds, thereby fostering positive health-related knowledge and behaviours [11–14]. Globally, the use of multimedia interventions for health promotion has gained traction, proving to be effective in disseminating information, influencing attitudes, and encouraging positive behaviour change [11, 14].

Despite these advancements, the existing body of literature reveals a critical gap pertaining to the specific impact and feasibility of MM-EE interventions in the unique socio-cultural and economic context of sub-Saharan Africa, particularly in South Africa. To address this gap, our study focuses on contributing novel insights into the effectiveness of MM-EE in enhancing FNL among community health workers (CHWs) who operate in resource-challenged South African townships, where obesity and diet-related health issues pose substantial challenges to public health [15, 16]. CHW is an encompassing term used to refer to a diverse group of lay health workers who provide healthcare services to communities and often do not have formal health education [17]. CHWs engage in a wide range of activities that contribute to multiple health programmes. Their responsibilities include: (i) promoting health and preventing illness; (ii) identifying and documenting health needs at the household level; (iii) offering psychosocial support; (iv) managing minor health conditions; (v) collaborating with other healthcare providers; (vi) supporting treatment adherence and providing counselling for chronic diseases; and (vii) ensuring continuity of care by identifying and reaching out to patients who have missed HIV and TB visits or require clinic referral [18, 19]. In addition, CHWs often reside in the townships where they work, ensuring shared socio-economic, cultural, and dietary experiences. This further implies shared languages, an important consideration as South Africa has eleven official languages. This similarity in lived experience promotes a deeper understanding of contextual factors that may influence FNL, and health in general. Community-based interventions involving CHWs have shown to be cost-effective and successful in Africa [17, 20, 21]. CHWs have proven to sometimes be more successful than clinical practitioners in providing health promoting activities, community education, social support, and behaviour change [22]. CHWs play a pivotal role in bridging the gap between healthcare services and communities, making them ideal agents for disseminating nutrition information and fostering behaviour change [23–25]. Examining the efficacy of MM-EE materials in the context of FNL among CHWs in particular holds significant potential for catalysing health improvement [23]. In townships where the burden of undernutrition among vulnerable segments of the population exists alongside an escalating prevalence of overweight and obesity, a pressing need for innovative and context-specific approaches has been identified [16, 26]. This therefore necessitates not only improving FNL but also empowering communities to make informed food choices within the tenets of food sovereignty.

By enhancing their FNL, this intervention study aims to equip frontline health workers to communicate nutrition-related information effectively to the communities they serve. While the study did not directly assess behaviour change outcomes, improved FNL support longer-term shifts in community-level nutrition behaviours [9]. This multimodal approach will allow for broader coverage, enabling CHWs to extend the reach of FNL and behaviour change communication to the community beyond face-to-face interactions, thus creating a ripple effect of positive health outcomes. The aim of this study was to investigate the effectiveness of MM-EE interventions in enhancing FNL among CHWs operating in resource-challenged South African townships.

## Methods

### Study design

This study forms part of a larger research project, Food Environments in Africa: Addressing Malnutrition using a Syndemics Approach (FoodSAMSA) [27], which aims to address malnutrition in all its forms by assessing its determinants and exploring interventions at macro- (policy), meso- (community) and micro- (interpersonal) level in South Africa. A quasi-experimental one-group pretest-post-test design was used for this study, ensuring that the outcome was measured in the same participants. A baseline assessment to capture participants’ initial FNL levels was conducted between July and August 2019, followed by an MM-EE intervention to enhance CHWs’ knowledge, perception and practice. A post-intervention assessment was conducted between February and March 2020.

### Study population and setting

This study was conducted among female CHWs aged 25 years and older (*n* = 96) who were employed by a non-profit organization and stationed in Kensington, Nyanga, and Gugulethu, three resource-limited townships in Cape Town, South Africa. These townships are known for their underdeveloped urban neighbourhoods where residents experience a poor quality of life because of multiple health and social challenges [28]. Gugulethu and Nyanga are predominantly Black townships that experience a combination of poverty, communicable and non-communicable diseases, a strong sense of community, and rapid development [29]. Their built environment consists mainly of shacks, brick and mortar houses for the wealthier individuals, sport fields, community centres, schools, and remains of former hostels [29]. They have electricity and running water and people typically share toilets and facilities [29]. Overpopulation in these townships causes strain to these services and constrained access to these resources [29]. Kensington has a population predominantly made up of people of mixed ancestry^1^. In comparison to Gugulethu or Nyanga, Kensington has a better economic stability, is less densely populated, and experiences lower levels of crime and poverty [30].

Of the 96 recruited participants, seven were lost to follow-up, and 12 were excluded from the final analysis due to discordant participant identification between pre- and post-intervention data, which included one participant with missing data in > 75% of datapoints. This resulted in a final sample of 77 participants. This nonetheless reflects a representative sample size for the three townships considering the WHO reported CHW density of 9.4 per 10,000 South African residents [31].

### Data collection

**Fig. 1:**
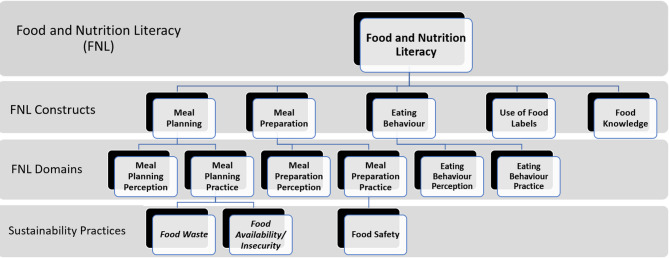
Conceptual Framework of MM-EE approach for CHWs. This framework outlines the five operational constructs used to assess food and nutrition literacy among community health workers: (i) Food knowledge, (ii) Meal planning, (iii) Meal preparation, (iv) Eating behaviours, and (v) Use of food labels. *Adapted from Vidgen & Gallegos (2014) and Nutbeam (2000)*

Data were collected at pre- and post-intervention using a structured questionnaire adapted from previously validated questionnaires and translated into Xhosa and Afrikaans, two local languages with validity ensured through post-translation pilot testing and support from the study investigators and CHW liaisons during survey [9, 32, 33]. Data were captured across the constructs: (i) Food knowledge, referring to understanding of food groups, key nutrients, and their health roles; (ii) Meal planning, defined as the ability to organise and budget for nutritionally balanced meals using available resources; (iii) Meal preparation, which encompassed basic cooking skills and safe food handling; (iv) Eating behaviours, describing patterns of intake such as portion control, frequency of meals, and snacking habits; and (v) Use of food labels, understood as the ability to read, interpret, and apply nutritional information on packaged foods to make informed choices. Each construct was defined and operationalised according to established FNL frameworks and literature [5, 7, 9]. The constructs were categorized in domains of literacy, including scientific knowledge, perception, and practice of nutrition (Fig. 1). The latter included subdomains of eating behaviour, meal planning and meal preparation. Surveys were paper based and conducted at the University of Western Cape, facilitated by the study investigators and three trained CHW liaisons. CHWs were collected from their work sites in the communities where they live and work, and transported to the campus. Individual travel expenses were not reimbursed, as logistical support was provided by the non-profit organisations managing the CHWs, which had formal agreements with the which had formal agreements with the School of Public Health at the University of Western Cape.

#### Informed consent

was obtained from participants before the start of the survey and responses were recorded electronically afterwards in an Excel spreadsheet by two different researchers.

### Measurements

We collected baseline information on demographic characteristics, education, employment, marital status, housing, and water sources. Body weight and height were measured using calibrated weighing scales and stadiometers, and body mass index (BMI) was calculated using the formula: weight in kilograms divided by height in metres squared (kg/m²). Underweight, normal weight, overweight, and obesity were defined using WHO-recommended BMI categories of < 18.5, 18.5–24.9, 25–29.9, and ≥ 30 kg/m², respectively [34]. Weight data were also collected at end-line for the majority of participants. However, given that the focus of this paper is on food and nutrition literacy outcomes, these anthropometric data will be reported in a separate study.

### Intervention implementation

MM-EE materials were disseminated over seven weeks between September and October 2019 among CHWs, who were encouraged to consult these materials in the next two months. In January 2020, the CHWs participated in the post-intervention survey and were interviewed using the same questionnaire administered during the baseline assessment (pre-intervention study).

The material conveyed food, nutrition, and health messages through entertaining short stories in the form of 2-page comic booklets and 30-second video clips. These materials, covering six topics, were developed and validated by nutrition experts in collaboration with graphic designers and media specialists, with detailed documentation available elsewhere (Mchiza et al., 2024 under review). The dissemination of these materials leveraged various media platforms, including Multimedia Messaging Service (MMS), Short Message Service (SMS), and WhatsApp messages with instructions on use attached. CHWs independently created WhatsApp groups to discuss the materials, enabling peer-to-peer engagement and informal learning. These discussions were not facilitated or analysed by the research team. Mobile data subscription was provided to participants to aid adherence.

### Statistical analysis

The survey contained Likert-type scales, consisting of four to six-level response categories. The Food knowledge construct was condensed to a binary response with responses coded as “correct” or “incorrect”. All other items were scored on a Likert scale where 1 indicated the least favourable score, 5 indicated the most favourable practice score, and 6 indicated the most favourable perception score. Negatively phrased questions were reverse-coded and items marked as ‘I don’t know’, or ’Never’ scored as 0. Multiple answers or unanswered items were treated as invalid responses and excluded. Missing data were replaced by the median scores for individual items where appropriate. Responses were computed in percentages and aggregated into composite scores for the different constructs, domains, and a composite FNL score using average scores of individual components. Participant scores were also standardized with categorization into “*Acceptable”* and “*Low”* levels of literacy was done using the threshold Z-score equal to or less than the mean of standardized scores, i.e., “0”.

Descriptive statistics, including mean, median, standard deviation, and frequency distributions, were used to summarize and describe the characteristics of the study participants. An Asset index variable was created using an unweighted principal component analysis of available assets [35]. Distribution of data was checked using boxplots and Q-Q Plots. Chi-square and Fishers’ Exact tests were used to evaluate categorical variables. Wilcoxon signed-rank test was used to analyse differences between groups. Multivariate linear regression using change scores (supplementary Table 1) and interaction terms was used to explore the relationship between participant characteristics and changes in FNL scores. Baseline sociodemographic data were used as dependent variables. To assess the effect of the intervention, a paired-sample analysis using *Cohen’s d* was used to estimate the effect sizes. Cohen’s d was interpreted as: 0.2 (small), 0.5 (moderate), and 0.8 (large) [36]. To account for testing multiple aggregated domains and subdomains, we applied the Benjamini-Hochberg procedure to adjust the *p*-values [37]. The analysis was conducted using R studio version 4.3.3. Significance was set at *p* <.05.

## Results

### Demographic and baseline characteristics

Baseline characteristics of the participants are shown in Table 1. Roughly 42% of the participants were between 35 and 44 years of age, with most participants having completed high school education, and having access to indoor water. It was further observed that over four-fifths (88.3%) of participants were classified as either being overweight or having obesity. Additionally, a significant number of the participants (68.8%) reported having an existing non-communicable disease (NCD). Baseline characteristics showed significant differences between subgroups (χ^2^: *p* = .00), except for marital status (χ^2^: *p* = .087). Fishers’ Exact test showed similar significant differences across study sites with regard to age (*p* = .02), water access (*p* <.00), and type of residence (*p* = .01).

**Table 1:** Baseline characteristics of the study population (*n* = 77)

**Demographics**	**P-value**		**Gugulethu **(***N* = 16)**	**Kensington **(***N* = 13)**	**Nyanga** (***N* = 48)**	**Total** (***N* = 77)**
**Age in years**		0.01*				
** 25–34**			0 (0%)	4 (30.8%)	8 (16.7%)	12 (15.6%)
** 35–44**			6 (37.5%)	3 (23.1%)	23 (47.9%)	32 (41.6%)
** 45–54**			6 (37.5%)	1 (7.7%)	12 (25.0%)	19 (24.7%)
** >55**			4 (25.0%)	5 (38.5%)	5 (10.4%)	14 (18.2%)
**Marital status**		0.09				
** Single**			10 (62.5%)	8 (61.5%)	28 (58.3%)	46 (59.7%)
** Married**			6 (37.5%)	5 (38.5%)	20 (41.7%)	31 (40.3%)
**Highest Level of Education**		0.00*				
** Primary**			0 (0%)	0 (0%)	7 (14.6%)	7 (9.1%)
** High school**			16 (100%)	12 (92.3%)	39 (81.3%)	67 (87.0%)
**Diploma or higher**			0 (0%)	1 (7.7%)	2 (4.2%)	3 (3.9%)
**Home Type**		0.00*				
** Shack**			7 (43.8%)	0 (0%)	22 (45.8%)	29 (37.7%)
** Brickhouse**			9 (56.3%)	11 (84.6%)	20 (41.7%)	40 (51.9%)
** Flat**			0 (0%)	2 (15.4%)	6 (12.5%)	8 (10.4%)
**Home water source**		0.00*				
** Communal tap**			1 (6.3%)	0 (0%)	11 (22.9%)	12 (15.6%)
** Outdoor tap**			6 (37.5%)	0 (0%)	9 (18.8%)	15 (19.5%)
** Indoor water**			9 (56.3%)	13 (100%)	28 (58.3%)	50 (64.9%)
**BMI Category**		0.00*				
** Underweight**			0 (0%)	1 (7.7%)	0 (0%)	1 (1.3%)
** Normal Weight**			3 (18.8%)	1 (7.7%)	3 (6.3%)	7 (9.1%)
** Overweight**			2 (12.5%)	4 (30.8%)	8 (16.7%)	14 (18.2%)
** Obese**			11 (68.8%)	7 (53.8%)	35 (72.9%)	54 (70.1%)
**Existing NCD**		0.00*				
** Absent**			6 (37.5%)	5 (38.5%)	13 (27.1%)	24 (31.2%)
** Present**			10 (62.5%)	8 (61.5%)	35 (72.9%)	53 (68.8%)

*Chi-square Goodness-of-fit test showing significant differences for subgroups

### Food and nutrition domains

**Fig. 2:**
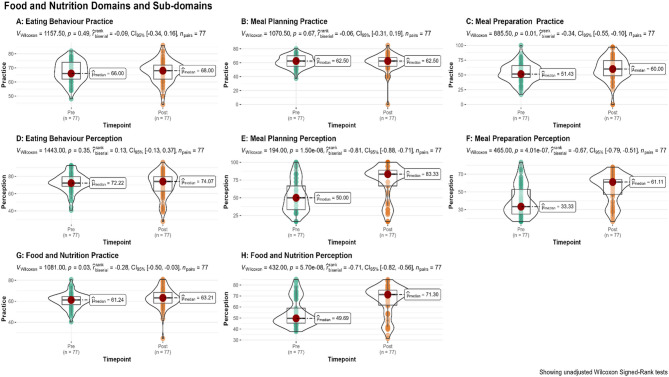
Violin box-plots showing pre- and post-score distribution for domains and subdomain

Pre- and post-intervention perception and practice scores of study participants are presented in Table 2; Fig. 2. For perception scores, significant improvements were observed across multiple subdomains. No significant change was observed in the eating behaviour subdomain median scores (pre-intervention: 72.2% [IQR: 11.1]; post-intervention: 74.1% [IQR: 16.7]; p.adj = 0.53). The total perception median score showed a significant improvement from 49.7% [IQR: 13.6] pre-intervention to 71.3% [IQR: 13.6] post-intervention (p.adj = 0.00). For practice scores, there was a significant improvement in the meal preparation subdomain, with median scores increasing from 51.4% [IQR: 20] pre-intervention to 60% [IQR: 25.7] post-intervention (p.adj = 0.02). The total practice median score showed a marginal increase from 61.2% [IQR: 7.6] to 63.2% [IQR:10.3] (p.adj = 0.05). A similar trend was also reported in the categorized levels as seen in supplementary Figs. 1 and 2.

**Table 2:** Pre-Post-intervention subdomain scores (*n* = 77)

	Practice scores Median (IQR)				Perception scores Median (IQR)		
Construct sub-domain	Pre-Intervention	Post-Intervention	*P*.adj value		Pre-Intervention	Post-Intervention	*P*.adj value
Eating Behaviour	66% (12)	68% (10)	0.60		72.2% (11.1)	74.1% (16.7)	0.53
Meal Preparation	51.4% (20)	60% (25.7)	0.02*		33.3% (27.8)	61.11% (16.7)	0.00*
Meal Planning	62.5% (12.5)	62.5% (15)	0.65		50% (33.3)	83.33% (22.2)	0.00*
Total	61.2% (7.6)	63.2% (10.3)	0.05		49.69% (13.6)	71.30% (13.6)	0.00*
*Significant Wilcoxon signed-rank test after correction for multiple testing							

### Food and nutrition literacy

No significant improvement was observed in eating behaviour across subgroups (Table 3). However, participants showed a substantial improvement in meal planning (Mdn = 58.6% [IQR: 12.1] pre- compared to 67.2% [IQR: 13.4] post-intervention; *p* <.001). The greatest improvement was observed among younger participants compared to those aged > 55 years. Both single and married individuals experienced significant gains, with individuals having a high school education showing more improvement than those with only a primary school education. Significant improvements were also observed in both participants with and without pre-existing NCDs. Those in the highest asset quintile (5th quintile) showed more marked improvement in meal planning compared to those in lower asset quintiles. Additionally, participants residing in Nyanga experienced significantly greater improvements in meal planning compared to those from Gugulethu and Kensington. Meal preparation also showed notable improvement (Mdn = 43.1% [ IQR: 19.6] pre- compared to 62.8% [ IQR: 11.7] post-intervention; *p* <.001). Individuals with obesity also showed significant improvement not only in meal planning and preparation but also in the use of food labels, food knowledge, and overall food literacy.

**Fig. 3:**
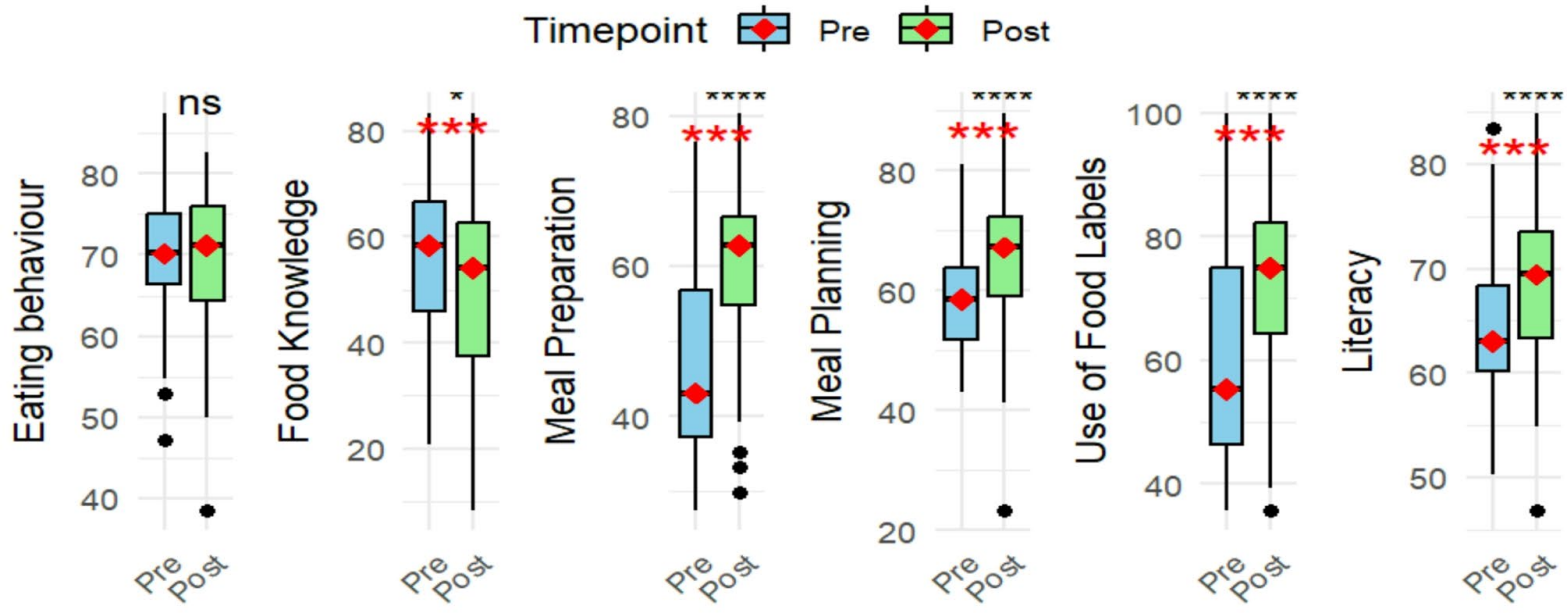
Food and Nutrition literacy constructs showing Pre- and Post-intervention scores

As seen in Fig. 3, a significant positive change was observed in the use of food labels, with the median score increasing from 55.4% [IQR: 28.6] pre- to 75.0% [ IQR: 17.9] post-intervention (*p* <.001). Food knowledge, however, showed a significant decrease from 58.3% [IQR: 28.6] pre- to 54.2% [IQR: 17.9] post-intervention (*p* <.05). This decrease in food knowledge was primarily seen among participants with pre-existing NCDs or obesity, and married individuals. Overall FNL median scores increased from 63.0 to 69.4% ([IQR: 8.1] to [IQR:10. 9], *p* = .00), suggesting an overall improvement in FNL. Improvements in literacy were particularly significant among participants aged 35–54, those with higher education, and individuals with obesity regardless of marital status. Significant predictors of positive change in literacy levels included residing in Nyanga (*p* <.05), and the interaction between obesity and pre-existing NCD (*p* = .05). A pre-existing NCD was, however, noted to be a significant predictor of negative change in FNL scores (*p* <.05) (Table 4).

The intervention had a moderate positive effect on overall FNL proficiency, as indicated by a Cohen’s d of 0.55. A similar positive effect was seen in food labelling proficiency (d = 0.58), meal planning proficiency (d = 0.49), and meal preparation proficiency, which showed the strongest positive effect (d = 0.70). These results demonstrate the intervention’s overall effectiveness in improving participants’ literacy and skills related to food and nutrition.

**Table 3:** Food and nutrition literacy constructs Pre- and Post-intervention showing sociodemographic distribution

Sociodemographics	Eating behaviour Median (IQR)		*p*-val	Meal planning Median (IQR)		*p*-val	Meal preparation Median (IQR)		*p*-val	Use of food labels Median (IQR)		*p*-val	Food knowledge Median (IQR)		*p*-val	Literacy Median (IQR)		*p*-val
	Pre	Post		Pre	Post		Pre	Post		Pre	Post		Pre	Post		Pre	Post	
**Age in years**																		
25–34	68.8 (7)	67.3(8.7)	1.00	52.6(10.8)	68.1(10.8)	0.01	48(18.6)	62.8(3.2)	0.01	46.4(19.6)	60.7(17.9)	0.06	56.2(21.9)	54.2(12.5)	0.31	62.5(6.3)	68.5(7.7)	0.11
35–44	70.2 (8.9)	69.2(13.5)	0.80	57.8(10.9)	67.2(18)	0.00	41.2(9.3)	62.8(22.1)	0.00	57.1(28.1)	75(14.3)	0.00	56.2(18.8)	43.8(20.8)	0.00	61.8(8.2)	69.1(10)	0.00
45–54	69.2 (9.6)	71.2(10.1)	0.70	60.3(9.1)	67.2(14.9)	0.05	41.2(8.8)	62.8(15.7)	0.00	60.7(27)	71.4(14.3)	0.05	58.3(16.7)	58.3(27.1)	0.62	63.8(6.7)	71(8.6)	0.00
>55	71.6 (10.3)	73.6(7)	0.68	62.1(17)	67.2(11.8)	0.62	49.5(24.5)	61.8(14.7)	0.35	57.1(24.6)	75(22.3)	0.04	60.4(19.8)	54.2(17.7)	0.31	65.6(14.9)	71.2(11)	0.46
**Marital status**																		
Single	71.2 (8.7)	69.2(11.1)	0.04	56.9(11.6)	67.2(15)	0.00	43.1(12.8)	62.8(14.7)	0.00	55.4(20.5)	71.4(20.5)	0.00	54.2(19.8)	50(16.7)	0.11	63.1(7.1)	67.1(10.1)	0.00
Married	69.2 (7.2)	72.1(10.1)	0.20	58.6(14.7)	67.2(11.2)	0.01	43.1(23.5)	64.7(8.8)	0.00	55.4(32.1)	75(12.5)	0.00	58.3(18.8)	54.2(25)	0.00	62.6(8.6)	71.3(6.2)	0.00
**Educational level**																		
Primary	74.0 (8.2)	68.3(9.1)	0.11	58.6(12.1)	67.2(15.3)	0.16	43.1(9.8)	62.8(13.2)	0.05	48.2(25)	78.6(33.9)	0.47	50(10.4)	54.2(16.7)	0.81	63.8(6.9)	70.9(10.9)	0.22
High school	70.2 (6.3)	71.2(12)	0.74	58.6(12.9)	67.2(13.6)	0.00	43.1(23.5)	62.8(11.8)	0.00	57.1(27)	75(17.9)	0.00	58.3(20.8)	54.2(25)	0.00	62.6(7.8)	69.4(10)	0.00
Diploma or higher	63.5 (11.1)	68.3(5.3)	0.50	54.3(7.8)	67.2(1.7)	0.37	39.2(12.8)	62.8(4.9)	0.25	46.4(19.6)	78.6(21.4)	1.00	54.2(8.3)	50(6.2)	0.37	64(9.4)	66.8(3)	0.25
**BMI Category** ^a^																		
Underweight	71.2 (0.0)	82.7(0)	1.00	67.2(0)	51.7(0)	1.00	66.7(0)	35.3(0)	1.00	75(0)	75(0)	0.00	70.8(0)	75(0)	1.00	74.3(0)	68.3(0)	1.00
Normal Weight	66.4 (12.5)	65.4(15.4)	0.93	60.8(11.2)	67.2(22.4)	0.94	45.1(21.6)	47.1(19.6)	0.83	71.4(32.1)	67.9(21.4)	0.69	41.7(20.8)	45.8(12.5)	1.00	62.4(9.6)	66.1(9.2)	0.81
Overweight	71.6 (15.1)	71.6(11.3)	0.71	58.2(17.5)	69.8(6.9)	0.06	44.1(25.5)	64.7(10.3)	0.12	53.6(13.4)	75(19.6)	0.12	68.8(24)	56.2(26)	0.05	65.1(13.8)	73.2(12.6)	0.16
Obese	70.2 (8.4)	71.2(10.6)	0.60	57.8(11.6)	67.2(12.9)	0.00	41.2(11.5)	62.8(11.8)	0.00	53.6(24.1)	75(14.3)	0.00	58.3(15.6)	52.1(19.8)	0.02	62.6(6.5)	69.3(8.8)	0.00
**Existing NCD**																		
Absent	70.7 (8.9)	72.1(11.3)	0.26	58.6(10.8)	68.1(12.7)	0.00	44.1(15.9)	63.7(6.9)	0.00	75(12.5)	60.7(28.6)	0.02	58.3(13.5)	54.2(16.7)	0.13	62.9(10.1)	72.3(8.8)	0.00
Present	70.2 (7.6)	70.2(11.5)	0.96	58.6(13.8)	67.2(17.2)	0.00	43.1(19.6)	62.8(19.6)	0.00	71.9(21.4)	53.6(25)	0.00	54.2(20.8)	50(25)	0.01	63(7.2)	68.3(10.2)	0.00
**Asset Index**																		
1st quintile	63.5 (6.7)	69.2(4.8)	0.75	77.6(7.8)	65.5(5.2)	0.37	74.5(7.8)	68.6(4.9)	1.00	78.6(5.4)	85.7(19.6)	1.00	62.5(16.7)	58.3(18.8)	0.50	76.6(4)	72.4(3.8)	1.00
2nd quintile	68.8 (7.0)	68.8(8.7)	1.00	52.2(6.9)	67.2(3.9)	0.09	41.2(8.3)	60.8(6.1)	0.44	71.4(15.2)	50(23.2)	0.42	54.2(18.8)	50(11.5)	0.42	60.9(4.9)	67.4(2.7)	0.16
3rd quintile	65.4 (9.9)	64.4(14.4)	0.40	51.7(7.8)	68.1(3)	0.09	44.1(6.4)	62.8(8.3)	0.06	66.1(16.1)	47.3(12.5)	0.09	58.3(6.2)	47.9(22.9)	0.44	61.2(7.4)	66.4(10.3)	0.09
4th quintile	69.2 (6.0)	69.2(6.2)	0.40	57.8(13.4)	62.9(16.8)	0.26	44.1(8.8)	58.8(19.1)	0.00	71.4(16.1)	58.9(22.6)	0.13	50(15.6)	52.1(24)	0.14	63.2(6.5)	65.5(8.5)	0.16
5th quintile	71.2 (7.2)	72.1(11.5)	0.73	58.6(11.4)	69(14.8)	0.00	42.2(22.6)	62.8(12.8)	0.00	75(16.1)	55.4(28.6)	0.00	58.3(21.9)	54.2(20.8)	0.03	63.2(8.2)	71.3(8.5)	0.00
**Location**																		
Kensington	71.2 (7.7)	75(8.7)	0.73	65.5(8.6)	65.5(13.8)	0.92	60.8(9.8)	64.7(9.8)	0.88	71.4(10.7)	53.6(26.8)	0.14	70.8(12.5)	58.3(12.5)	0.25	72.1(10.6)	72.4(5.7)	0.97
Nyanga	71.2 (6.7)	70.2(9.9)	0.18	53.9(10.3)	67.2(14.7)	0.00	39.2(7.8)	62.8(20.1)	0.00	75(21.4)	50(17.9)	0.00	54.2(17.7)	50(25)	0.03	61.5(3.9)	67.7(10)	0.00
Gugulethu	66.4 (4.3)	69.7(16.1)	0.65	69(15.5)	72.4(5.6)	0.28	60.8(21.6)	64.7(12.8)	0.39	75(15.2)	71.4(27.7)	0.27	58.3(17.7)	45.8(30.2)	0.05	67.4(8.7)	70.8(7.9)	0.38
**Total**	70.2(8.7)	71.2(11.5)	**0.41**	58.6(12.1)	67.2(13.4)	0.00*	43.1(19.6)	62.8(11.7)	0.00*	55.4(28.6)	75.0(17.9)	0.00*	58.3(28.6)	54.2(17.7)	0.00*	63.0(8.1)	69.4(10.9)	0.00*

*Significant Wilcoxon signed-rank test after correction for multiple testing

**Table 4:** Multivariable analysis for percentage change in FNL scores (*n* = 77)

Predictor	Beta Coefficient (β)	95% (CI)	*p*-value	
**Intercept**	16.51	(–2.48, 35.49)	0.09	
**Obesity**	− 12.11	(–29.26, 5.04)	0.17	
**Overweight**	6.31	(–1.32, 13.94)	0.11	
**Underweight**	− 1.60	(–9.32, 6.12)	0.86	
**Age25-34**	− 1.37	(–9.02, 6.26)	0.87	
**Age35-44**	− 5.91	(–14.70, 2.88)	0.18	
**Age45-54**	− 5.09	(–13.26, 3.08)	0.22	
**Nyanga**	7.67	(1.71, 13.23)	0.01*	
**Gugulethu**	0.66	(–6.17, 7.50)	0.85	
**Pre-existing NCD**	− 29.97	(–50.41, − 9.54)	0.00*	
**Obesity & Pre-existing NCD**	18.53	(–0.33, 37.40)	0.05*	
**Age 25–34 & Pre-existing NCD**	8.47	(–10.58, 27.53)	0.37	
**Age 35–44 & Pre-existing NCD**	14.94	(4.61, 25.27)	0.01*	
**Age 45–54 & Pre-existing NCD**	13.93	(2.85, 25.03)	0.02*	

* Significant predictors of change in Literacy levels

## Discussion

This study highlights the effectiveness of an MM-EE intervention designed to enhance FNL among CHWs from resource-limited townships in Cape Town, South Africa. The intervention led to significant improvements in meal planning, meal preparation, and the use of food labels, although eating behaviour did not show significant improvement. The FNL perception domain also showed marked improvement. Significant improvement was only seen in the meal planning practice subdomain for the FNL practice domain. These findings align with existing research, in which knowledge and skills-based improvements did not always correlate with behaviour change, and highlight the complexity of behaviour change in public health nutrition interventions [38, 39]. While participants may learn practical skills, they may not fully understand the underlying principles or have the resources to consistently apply them in their daily lives, particularly in resource-constrained environments [40]. This disconnect between knowledge acquisition and sustained behaviour change is well-documented in the public health nutrition literature. While interventions often succeed in imparting practical skills, participants may not develop a deep understanding of the underlying nutritional principles, which is essential for long-term adoption of healthy practices [9]. Furthermore, the ability to consistently apply these skills is frequently constrained by external factors such as limited access to healthy foods, financial barriers, and competing household priorities, particularly in low-resource settings [9, 41, 42]. These contextual challenges can undermine the translation of knowledge and skills into actual dietary behaviour change, underscoring the need for interventions that address both individual competencies and broader environmental supports.

The significant improvement in meal planning, particularly among younger participants and those with lower educational attainment, is consistent with the literature suggesting that younger individuals are more responsive to nutrition interventions. Research indicates that younger adults tend to adopt new dietary behaviours more readily, as they are in a life-phase where forming habits is more dynamic compared to older individuals [43, 44]. The intervention’s effectiveness on practical meal-related skills suggests that interventions focusing on developing cooking and planning competencies can be particularly effective in populations with limited resources and FNL [45]. The significant changes in meal planning perception also reflect the success of educational components that emphasize the importance of structured meal planning in promoting healthier eating habits. This aligns with findings that suggest education targeting meal organization and preparation can enhance individuals’ confidence and attitudes toward meal planning, even when immediate behaviour change is not observed [46].

Contrary to expectations, participants’ food knowledge demonstrated a significant decline post-intervention. This unexpected outcome raises questions regarding the potential bias involved or unintended consequences of MM-EE intervention. This outcome may reflect not only the content and delivery of the intervention but also broader systemic challenges, including the high expectations placed on CHWs. Several other factors may have contributed to the post-intervention decline. Evidence suggests that when interventions are multifaceted or overly complex, CHWs can become overwhelmed, which may hinder their ability to fully comprehend, retain, and apply new information [47]. Interventions that introduce multiple components simultaneously can be challenging, particularly if adequate support or guidance is not provided [45], and as the study was interrupted by the onset of the Covid-19 pandemic, this might be the case. Secondly, several food items in the questionnaire may not be commonly consumed by participants in resource-limited townships. While participants may have become more adept at practical tasks (like meal preparation and label reading), there may have been a disconnect in translating knowledge to practice. Sociodemographic factors, such as the level of education, age, and asset index quintile which connotes participants’ socioeconomic disparities, limited access to resources, and cultural differences can also impact individuals’ ability to comprehend, retain and apply nutritional information, especially pertaining to the physiobiological knowledge of food [48, 49]. The effectiveness of nutrition training for CHWs is closely linked to the relevance, accessibility, and adaptability of the training materials to their specific needs and educational backgrounds. While the recent South Africa National Department of Health (NDOH) 2024 training package represents a positive step towards standardising CHW training, it is important to note that this package does not currently provide a detailed framework or comprehensive guidelines specifically for FNL training [19]. This gap is consistent with broader findings in South Africa, where existing training frameworks for nutrition-related roles often lack comprehensive coverage of food literacy and practical competencies, resulting in variable knowledge gains and skills among frontline workers. Addressing such omissions, alongside challenges such as heavy workloads and limited resources, is critical for equipping CHWs to deliver effective nutrition education and for improving health outcomes in the communities they serve.

The observed negative association between pre-existing NCDs and changes in FNL underscores the unique challenges faced by CHWs living with chronic health conditions. Studies have shown that individuals with NCDs often have lower baseline health literacy, and improving these levels may require more tailored and intensive interventions [50, 51]. Several studies have highlighted barriers such as inadequate training, high workload, and insufficient support for CHWs delivering NCD services, emphasizing the need for tailored training and system-level interventions [47, 52]. While the literature does not specifically address screening and counselling CHWs with NCDs prior to deployment, integrating such occupational health measures could further strengthen the frontline work force. Responsibility for such screening and support should rest with the employing health authorities, such as the NDOH or local health districts, which are best positioned to integrate these services into existing occupational health frameworks. While age groups did not significantly predict changes in literacy scores, when paired with reported history of NCDs, significant positive literacy scores were observed among older age groups. This may be explained by the fact that older age groups might have been more serious about improving their literacy. Local research suggests that older individuals are more aware of their health status if they have been diagnosed with an NCD, are enrolled in treatment, and are educated on the risk of complications/mortality from uncontrolled NCDs [53, 54], hence an increased motivation to adopt healthier lifestyles. Study results also indicated that participants from one study site (Nyanga) had an eight-fold higher chance of experiencing positive changes in FNL, asserting that contextual factors play a critical role in the success of MM-EE interventions and that location-specific tailoring is essential to address the unique challenges and opportunities faced by different communities [55].

Baseline BMI was not significantly associated with changes in FNL over the intervention period, suggesting that initial body weight status did not influence the intervention’s impact on FNL outcomes. While studies have shown a negative correlation between literacy levels and overweight/obesity [56, 57], the significance of obesity as predictor of changes in health literacy and behaviour is still inconclusive [58]. However, the borderline significance of the interaction between obesity and NCDs suggests that obesity may modify the relationship between NCDs and literacy changes. Obesity is often associated with increased health risks, and individuals with both obesity and NCDs may benefit from specific literacy interventions aimed at mitigating these risks [59]. However, the findings from this research align with studies from low- and middle-income countries that indicate obesity alone may not significantly affect health literacy outcomes when contextual factors like NCD prevalence and socio-economic status are not considered [60].

The overall improvement in FNL suggests that the intervention was effective in enhancing participants’ practical competencies related to food choices, preparation, and label use. The moderate positive effect sizes for overall literacy proficiency and specific constructs such as meal planning, meal preparation, and food label use underscore the intervention’s effectiveness, and further explain the overall improvement in FNL despite the unexpected decline in food knowledge. These effect sizes fall within the range typically seen in similar FNL interventions, further validating the intervention’s effectiveness [36]. The use of a culturally tailored quasi-experimental design allowed for an effective evaluation of changes in FNL over time within the same group of participants. This is particularly advantageous in real-world settings where randomization is not feasible, and further supports the evidence of the intervention’s effectiveness [61]. A study by Resnicow et al. [62] has shown that interventions tailored to cultural contexts are more effective in promoting behaviour change as they resonate better with the target audience [62]. Additionally, the innovative use of entertainment-education materials, such as comic booklets and short video clips, likely played a crucial role in sustaining participant interest and engagement throughout the intervention period [11]. The provision of internet data for viewing this material compensated for resource constraints. Finally, by enhancing their FNL, the study not only benefitted CHWs directly, but also has the potential for a broader community impact through knowledge dissemination as engaging CHWs in health interventions can lead to more sustainable outcomes [63].

The study also had some limitations. While efforts were made to design robust measurement tools and use methodological rigour, a nonblinded, peer-learning WhatsApp group where participants could discuss the intervention could have resulted in some bias of study results. For example, shared information bias may have led participants to focus discussions on familiar topics, overlooking less intuitive or nuanced content [64]. Additionally, social influence bias within group settings might have encouraged members to conform to dominant views or commonly held beliefs. Use of the MM-EE material was also not directly observed, and the implementation and post-intervention were interrupted by the Covid-19 pandemic contributing to socioeconomic and other health challenges for study participants [65].

## Conclusion

The MM-EE intervention described in this study demonstrated effectiveness in improving FNL among CHWs in South Africa. This study provides further evidence on the importance of context-specific adaptations in public health interventions and the need for ongoing support of education interventions to reinforce knowledge and translate it into sustainable behaviour change. While significant improvements were observed in practical literacy skills, the gaps in food knowledge highlight the need for interventions to be carefully designed to address all dimensions of literacy. The influence of sociodemographic factors further emphasizes the necessity of tailored approaches in health education. Future research should investigate the mechanisms underlying the contradictory findings, particularly the decline in food knowledge scores, to enhance the effectiveness of similar interventions in comparable settings.

## Electronic supplementary material

Below is the link to the electronic supplementary material.

Supplementary Material 1

## Data Availability

Data used in this study will be made available to interested parties and the journal on request. To obtain access to the raw data analysed in your study kindly contact the corresponding author; Elochukwu C. Okanmelu at email address; elochukwu.okanmelu@ibe.med.uni-muenchen.de.

## Footnotes

1 In the South African context, the term “Coloured” refers to a legally recognized demographic category historically used to describe people of mixed racial ancestry. Although this classification continues to be used in official government reporting and policy frameworks, it may be considered inappropriate or misunderstood in international contexts. In this manuscript, we use “people of mixed ancestry” for clarity and cultural sensitivity [1,2].

## References

[1] Gibbs HD, Ellerbeck EF, Gajewski B, Zhang C, Sullivan DK. The nutrition literacy assessment instrument is a valid and reliable measure of nutrition literacy in adults with chronic disease. J Nutr Educ Behav. 2018;50(3):247–e571. 29246567 10.1016/j.jneb.2017.10.008 PMC5845801

[2] Fernandez RM. SDG3 Good health and Well-Being: Integration and Connection with Other SDGs. In: Leal Filho W, Wall T, Azul AM, Brandli L, Özuyar PG, editors. Good health and Well-Being. Cham: Springer International Publishing. 2020;629–36.

[3] Popkin BM, Corvalan C, Grummer-Strawn LM. Dynamics of the double burden of malnutrition and the changing nutrition reality. Lancet. 2020;395(10217):65–74. 31852602 10.1016/S0140-6736(19)32497-3 PMC7179702

[4] Vettori V, Lorini C, Milani C, Bonaccorsi G. Towards the Implementation of a Conceptual Framework of Food and Nutrition Literacy: Providing Healthy Eating for the Population. International Journal of Environmental Research and Public Health. 2019;16(24). 10.3390/ijerph16245041 PMC6950737 31835678

[5] Krause C, Sommerhalder K, Beer-Borst S, Abel T. Just a subtle difference? Findings from a systematic review on definitions of nutrition literacy and food literacy. Health Promot Int. 2018;33(3):378–89. 27803197 10.1093/heapro/daw084 PMC6005107

[6] Nutbeam D. Health literacy as a public health goal: a challenge for contemporary health education and communication strategies into the 21st century. Health Promot Int. 2000;15(3):259–67.

[7] Truman E, Lane D, Elliott C. Defining food literacy: A scoping review. Appetite. 2017;116:365–71. 28487244 10.1016/j.appet.2017.05.007

[8] Conrad Z, Niles MT, Neher DA, Roy ED, Tichenor NE, Jahns L. Relationship between food waste, diet quality, and environmental sustainability. PLoS ONE. 2018;13(4):e0195405. 29668732 10.1371/journal.pone.0195405 PMC5905889

[9] Vidgen HA, Gallegos D. Defining food literacy and its components. Appetite. 2014;76:50–9. 24462490 10.1016/j.appet.2014.01.010

[10] Silva P, Araújo R, Lopes F, Ray S. Nutrition and food literacy: framing the challenges to health communication. Nutrients. 2023;15(22). 10.3390/nu15224708 PMC10674981 38004102

[11] Singhal A, Rogers EM. A theoretical agenda for Entertainment-Education. Communication Theory. 2006;12(2):117–35.

[12] Hieftje K, Edelman EJ, Camenga DR, Fiellin LE. Electronic media-based health interventions promoting behavior change in youth: a systematic review. JAMA Pediatr. 2013;167(6):574–80. 23568703 10.1001/jamapediatrics.2013.1095 PMC3733329

[13] Chagas C, Melo GR, Botelho RBA, Toral N. Effects of the Rango cards game intervention on food consumption, nutritional knowledge and self-efficacy in the adoption of healthy eating practices of high school students: a cluster randomised controlled trial. Public Health Nutr. 2020;23(13):2424–33. 32476640 10.1017/S1368980020000531 PMC11374568

[14] Jacobs RJ, Lou JQ, Ownby RL, Caballero J. A systematic review of eHealth interventions to improve health literacy. Health Inf J. 2016;22(2):81–98. 10.1177/1460458214534092 24916567

[15] Roomaney RA, van Wyk B, Cois A, Pillay-van Wyk V. Inequity in the distribution of Non-Communicable disease Multimorbidity in adults in South africa: an analysis of prevalence and patterns. Int J Public Health. 2022;67:1605072. 36051505 10.3389/ijph.2022.1605072 PMC9426027

[16] Ramalivhana FW, Veldsman T, Moss SJ. Assessment of non-communicable disease risk factors, functional performance, and health-related quality of life in adults: a comparative analysis in low-resourced urban and rural areas of South Africa. BMC Public Health. 2024;24(1):1580. 38867182 10.1186/s12889-024-18964-2 PMC11170915

[17] van Ginneken N, Lewin S, Berridge V. The emergence of community health worker programmes in the late apartheid era in South africa: an historical analysis. Soc Sci Med. 2010;71(6):1110–8. 20638169 10.1016/j.socscimed.2010.06.009 PMC2941026

[18] South African National Department of Health. Policy framework and strategy for Ward-Based primary healthcare outreach teams 2018/19–2023/24. Pretoria: South African National Department of Health. 2018;22.

[19] South African National Department of Health. Participant manual 10.2: integrated final. Pretoria: National Department of Health. 2024.

[20] Tsolekile LP, Puoane T, Schneider H, Levitt NS, Steyn K. The roles of community health workers in management of non-communicable diseases in an urban Township. Afr J Prim Health Care Fam Med. 2014;6(1):E1–8. 26245419 10.4102/phcfm.v6i1.693 PMC4565048

[21] Egbujie BA, Delobelle PA, Levitt N, Puoane T, Sanders D, van Wyk B. Role of community health workers in type 2 diabetes mellitus self-management: A scoping review. PLoS ONE. 2018;13(6):e0198424. 29856846 10.1371/journal.pone.0198424 PMC5983553

[22] Salomé Kruger H. Community health workers can play an important role in the prevention and control of non-communicable diseases in poor communities. Taylor & Francis 2006;52–4.

[23] Braun R, Catalani C, Wimbush J, Israelski D. Community health workers and mobile technology: a systematic review of the literature. PLoS ONE. 2013;8(6):e65772. 23776544 10.1371/journal.pone.0065772 PMC3680423

[24] Kangovi S, Mitra N, Norton L, Harte R, Zhao X, Carter T, et al. Effect of community health worker support on clinical outcomes of Low-Income patients across primary care facilities: A randomized clinical trial. JAMA Intern Med. 2018;178(12):1635–43. 30422224 10.1001/jamainternmed.2018.4630 PMC6469661

[25] Grant M, Wilford A, Haskins L, Phakathi S, Mntambo N, Horwood CM. Trust of community health workers influences the acceptance of community-based maternal and child health services. Afr J Prim Health Care Family Med. 2017;9(1):1–8. 10.4102/phcfm.v9i1.1281 PMC5458568 28582988

[26] Nel JH, Steyn NP. The nutrition transition and the double burden of malnutrition in Sub-Saharan African countries: how do these countries compare with the recommended LANCET COMMISSION global diet?? Int J Environ Res Public Health. 2022;19(24). 10.3390/ijerph192416791 PMC9779835 36554669

[27] Food environments in AFrica. Addressing Malnutrition using a Syndemic Approach 2024 [updated September 2024; cited 2024 14, September]. Available from: https://foodsamsa.samrc.ac.za/

[28] Moghayedi A, Mehmood A, Michell K, Ekpo CO. Modeling the neighborhood wellbeing of townships in South Africa. Sustainability. 2023;15(11):8542.

[29] Smith K. The status of cape town: development overview. Cape Town: City of Cape Town & Islandia Institute; 2005 https://www.isandla.org.za/en/projects/urban-development/item/download/49_a45a37b753eaa7a4d6fd9014676f9fda

[30] Spatial Data Infrastructure and Geographic Information Systems, Departments C. OF CAPE TOWN. City of Cape Town–2011 Census Suburb Kensington. 2013.

[31] World Health Organisation. The state of the health workforce in the who African region. Brazzaville, Republic of Congo: World Health Organization, Regional Office for Africa. 2021.

[32] Vorster HH, Badham J, Venter C. An introduction to the revised food-based dietary guidelines for South Africa. South Afr J Clin Nutr. 2013;6:S1–164.

[33] Ketelo A. Determining food and nutrition literacy of community health workers in the Western Cape, South Africa. 2020.

[34] World Health Organisation. Physical status: the use and interpretation of anthropometry. Report of a WHO Expert Consultation. WHO Technical Report Series Number 854. World Health Organization Geneva. 1995. 8594834

[35] Rutstein S, Johnson K. The DHS Wealth Index 2004.

[36] Cohen J. Statistical power analysis for the behavioral sciences. routledge 2013.

[37] Benjamini Y, Hochberg Y. Controlling the false discovery rate: A practical and powerful approach to multiple testing. J Roy Stat Soc: Ser B (Methodol). 1995;57(1):289–300.

[38] McCluskey A, Lovarini M. Providing education on evidence-based practice improved knowledge but did not change behaviour: a before and after study. BMC Med Educ. 2005;5(1):40. 16364181 10.1186/1472-6920-5-40 PMC1352357

[39] Tengland PA. Behavior change or empowerment: on the ethics of Health-Promotion goals. Health Care Anal. 2016;24(1):24–46. 24100936 10.1007/s10728-013-0265-0

[40] Hashemiparast M, Negarandeh R, Theofanidis D. Exploring the barriers of utilizing theoretical knowledge in clinical settings: A qualitative study. Int J Nurs Sci. 2019;6(4):399–405. 31728392 10.1016/j.ijnss.2019.09.008 PMC6838863

[41] Fisher HJH, Erasmus AC, Viljoen AT. Developing a food literacy definition for South Africa. 2019.

[42] Contento IR. Nutrition education: linking research, theory, and practice. 2007;17–25. 18296331

[43] Stok FM, Renner B, Clarys P, Lien N, Lakerveld J, Deliens T. Understanding eating behavior during the transition from adolescence to young adulthood: A literature review and perspective on future research directions. Nutrients. 2018;10(6). 10.3390/nu10060667 PMC6024552 29794986

[44] Poobalan AS, Aucott LS, Clarke A, Smith WC. Diet behaviour among young people in transition to adulthood (18–25 year olds): a mixed method study. Health Psychol Behav Med. 2014;2(1):909–28. 25750826 10.1080/21642850.2014.931232 PMC4346025

[45] Slater J, Falkenberg T, Rutherford J, Colatruglio S. Food literacy competencies: A conceptual framework for youth transitioning to adulthood. Int J Consumer Stud. 2018;42.

[46] Roy R, Kelly B, Rangan A, Allman-Farinelli M. Food Environment Interventions to Improve the Dietary Behavior of Young Adults in Tertiary Education Settings: A Systematic Literature Review. J Acad Nutr Diet. 2015;115(10):1647-81.e1. 10.1016/j.jand.2015.06.380 26271691

[47] Odendaal WA, Anstey Watkins J, Leon N, Goudge J, Griffiths F, Tomlinson M, et al. Health workers’ perceptions and experiences of using mHealth technologies to deliver primary healthcare services: a qualitative evidence synthesis. Cochrane Database Syst Rev. 2020;3(3):Cd011942. 32216074 10.1002/14651858.CD011942.pub2 PMC7098082

[48] Gittelsohn J, Trude A. Diabetes and obesity prevention: changing the food environment in low-income settings. Nutr Rev. 2017;75(suppl1):62–9. 28049750 10.1093/nutrit/nuw038 PMC5207007

[49] Sinclair S, Hammond D, Goodman S. Sociodemographic differences in the comprehension of nutritional labels on food products. J Nutr Educ Behav. 2013;45(6):767–72. 23886777 10.1016/j.jneb.2013.04.262

[50] Zarcadoolas C, Pleasant A, Greer DS. Understanding health literacy: an expanded model. Health Promot Int. 2005;20(2):195–203. 15788526 10.1093/heapro/dah609

[51] Jeet G, Thakur J, Prinja S, Singh M. Community health workers for non-communicable diseases prevention and control in developing countries: evidence and implications. PLoS ONE. 2017;12. 10.1371/journal.pone.0180640 PMC5509237 28704405

[52] Rawal L, Jubayer S, Choudhury SR, Islam SMS, Abdullah AS. Community health workers for non-communicable diseases prevention and control in bangladesh: a qualitative study. Glob Health Res Policy. 2020;6(1):1. 33407942 10.1186/s41256-020-00182-z PMC7786185

[53] Stokes A, Berry KM, McHiza Z, Parker WA, Labadarios D, Chola L, et al. Prevalence and unmet need for diabetes care across the care continuum in a National sample of South African adults: evidence from the SANHANES-1, 2011–2012. PLoS ONE. 2017;12(10):e0184264. 28968435 10.1371/journal.pone.0184264 PMC5624573

[54] Ohene-Kwofie D, Riumallo-Herl C, Kabudula C, Gómez-Olivé FX. Sociodemographic disparities in awareness of chronic conditions: an observational study among older persons in rural north-east of South Africa. BMJ Public Health. 2024;2(1):e000315. 40018210 10.1136/bmjph-2023-000315 PMC11812760

[55] Vijayakumar NP, Neally SJ, Potharaju KA, Curlin K, Troendle JF, Collins BS et al. Customizing Place-Tailored Messaging Using a Multilevel Approach: Pilot Study of the Step It Up Physical Activity Mobile App Tailored to Neighborhood Environment. Circulation: Cardiovascular Quality and Outcomes. 2022;15(11):e009328. 10.1161/CIRCOUTCOMES.122.009328 PMC9680010 36378765

[56] Toçi E, Burazeri G, Kamberi H, Toçi D, Roshi E, Jerliu N, et al. Health literacy and body mass index: a population-based study in a South-Eastern European country. J Public Health. 2021;43(1):123–30. 10.1093/pubmed/fdz103 31768531

[57] Adewole KO, Ogunfowokan AA, Olodu M. Influence of health literacy on health promoting behaviour of adolescents with and without obesity. Int J Afr Nurs Sci. 2021;15:100342.

[58] Cheong SM, Mohamad Nor NS, Ahmad MH, Manickam M, Ambak R, Shahrir SN, et al. Improvement of health literacy and intervention measurements among low socio-economic status women: findings from the mybff@home study. BMC Womens Health. 2018;18(Suppl 1):99. 30066659 10.1186/s12905-018-0596-y PMC6069691

[59] Berkman ND, Sheridan SL, Donahue KE, Halpern DJ, Crotty K. Low health literacy and health outcomes: an updated systematic review. Ann Intern Med. 2011;155(2):97–107. 21768583 10.7326/0003-4819-155-2-201107190-00005

[60] Lago - Peñas S, Rivera B, Cantarero D, Casal B, Pascual M, Blázquez-Fernández C, et al. The impact of socioeconomic position on non-communicable diseases: what do we know about it? Perspect Public Health. 2021;141(3):158–76. 32449467 10.1177/1757913920914952

[61] Cook TD, Campbell DT. Causal inference and the language of experimentation. Quasi-experimentation: Design & analysis issues for field settings. 1979:1–36.

[62] Resnicow K, Soler R, Braithwaite RL, Ahluwalia JS, Butler J. Cultural sensitivity in substance use prevention. J Community Psychol. 2000;28(3):271–90.

[63] Olaniran A, Smith H, Unkels R, Bar-Zeev S, van den Broek N. Who is a community health worker? - a systematic review of definitions. Glob Health Action. 2017;10(1):1272223. 28222653 10.1080/16549716.2017.1272223 PMC5328349

[64] Pasquetto I, Jahani E, Atreja S, Baum M. Social Debunking of Misinformation on WhatsApp: The Case for Strong and In-group Ties. Proceedings of the ACM on Human-Computer Interaction. 2022;6:1–35.

[65] Marchetti-Mercer MC, Laher S, Watermeyer J, Hassem T. Loadshedding, safety concerns, and mental health in South Africa. South Afr J Psychol. 2024;54(3):415–20.

