# Supplementary material for: Supporting community health workers in South Africa for context-specific food and nutrition literacy: implementation of a multi-media education-entertainment intervention
Source: BMC Nutr. 2025 Jul 25;11:144. doi: 10.1186/s40795-025-01124-z (PMC12291494; doi:10.1186/s40795-025-01124-z)
Supplement: Supplementary file 1 — Supplementary Material 1 [file 40795_2025_1124_MOESM1_ESM.docx]

**Supplementary Material**

**Supplementary material to:** Elochukwu C. Okanmelu, Machoene D. Sekgala, Peter Delobelle, Olufunke Alaba, Nicole Holliday, Jillian Hill, Martina Lembani, Zandile J. Mchiza. Supporting community health workers for context-specific food and nutrition literacy: the implementation of a multi-media edutainment intervention for South Africans.

**Corresponding author:**

Elochukwu C. Okanmelu, MD, MHE, MSc, Chair of Public Health and Health Services Research, Institute for Medical Information Processing, Biometry and Epidemiology (IBE), Ludwig-Maximilians-Universität (LMU Munich), Elisabeth-Winterhalter-Weg 6, D-81677 Munich, Germany, elochukwu.okanmelu@ibe.med.uni-muenchen.de. Phone: +49 174 866 7125

1. Supplementary figure 1: Categorized FNL Constructs


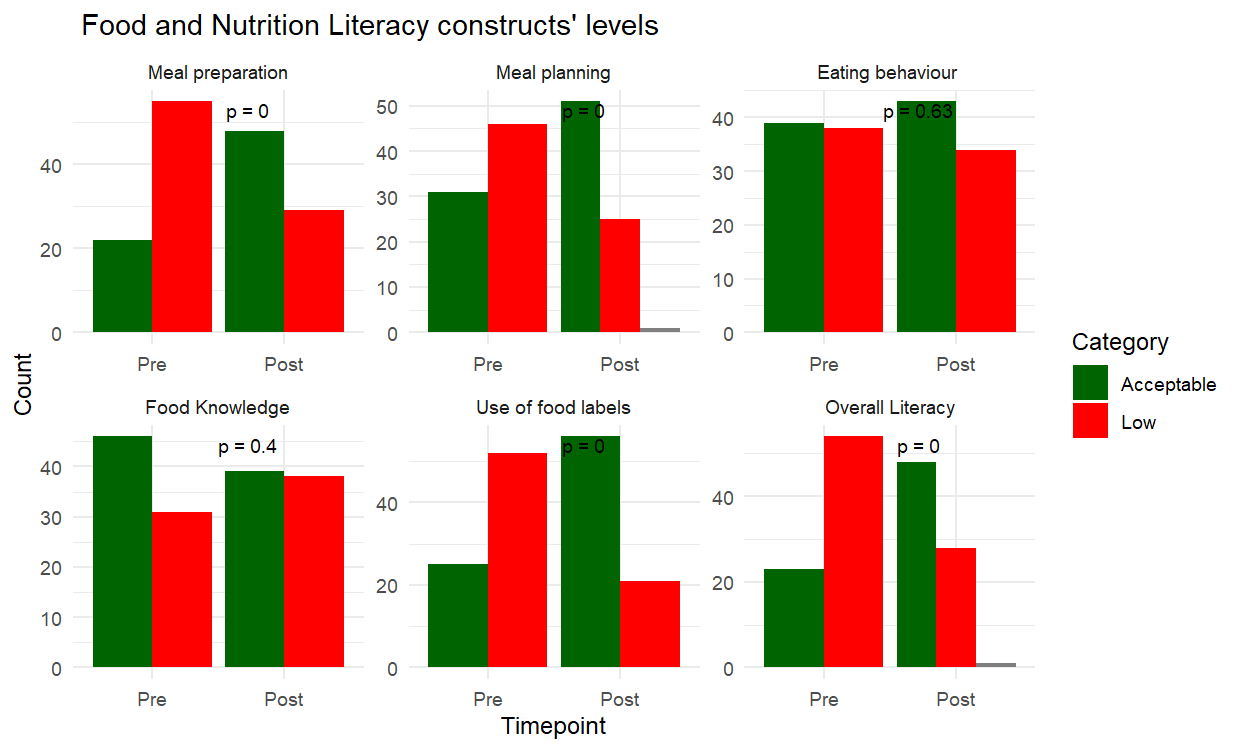


1. Supplementary figure 2: showing categorized FNL domains and sub-domains


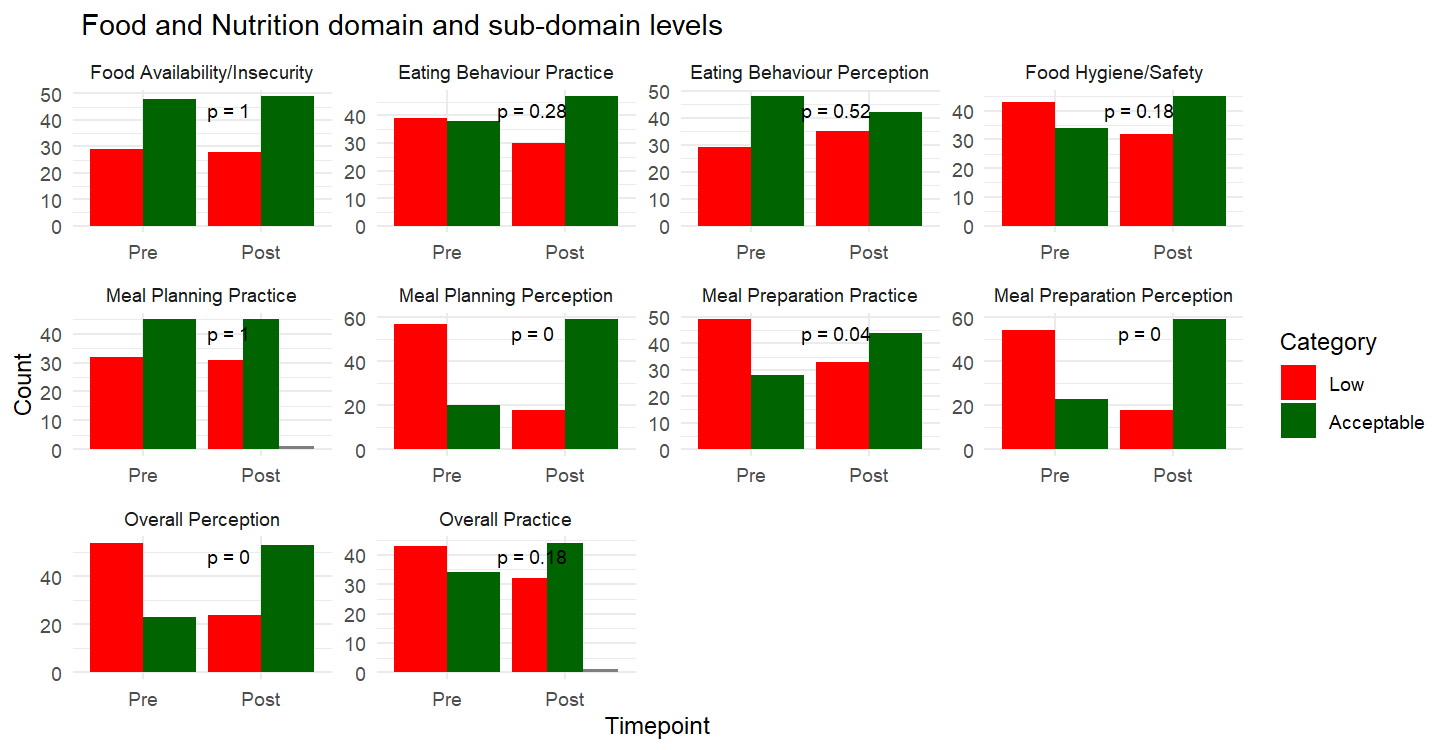


1. Supplementary Table 1: FNL and FNL Constructs’ Change Scores

| **Change scores for constructs** | | | | | | |
| --- | --- | --- | --- | --- | --- | --- |
|  | **Eating Behaviour** | **Food Knowledge** | **Meal Preparation** | **Meal Planning** | **Use of Food Label** | **Overall Literacy** |
| Mean (SD) | -0.99(8.69) | -6.11(15.13) | 11.99(20.09) | 8.49(17.60) | 11.74(20.27) | 4.90(8.73) |
